# The impact of body mass index on short-term and long-term surgical outcomes of laparoscopic hepatectomy in liver carcinoma patients: a retrospective study

**DOI:** 10.1186/s12957-022-02614-1

**Published:** 2022-05-10

**Authors:** Lei Zhao, Jiangang Wang, Jingxia Kong, Xing Zheng, Xin Yu

**Affiliations:** 1 Department of Anesthesiology, Sir Run Run Shaw Hospital, School of Medicine, Zhejiang University, Hangzhou, 310016 China; 2 Hangzhou Emergency Medical Center, Hangzhou, 310021 Zhejiang China; 3 Department of Investment and Insurance, Zhejiang Financial College, Hangzhou, Zhejiang Province China

**Keywords:** Body mass index, Liver carcinoma, Laparoscopic Hepatectomy, Risk factors, Long-term outcome, underweight patients with liver carcinoma.

## Abstract

**Background:**

It was generally accepted that obesity could increase the morbidity and mortality of surgical patients. However, the influence of body mass index (BMI) on short-term and long-term surgical outcomes of laparoscopic hepatectomy (LH) for patients with liver carcinoma remains unclear. The aim of this study was to evaluate the influence of BMI on surgical outcomes.

**Methods:**

From August 2003 to April 2016, 201 patients with liver carcinoma who underwent LH were enrolled in our study. Based on their BMI in line with the WHO’s definition of obesity for the Asia-Pacific region, patients were divided into three groups: underweight (BMI< 18.5 kg/m^2^), normal weight (18.5≤BMI< 23 kg/m^2^), and overweight (BMI≥ 23 kg/m^2^). Demographics and surgical outcomes of laparoscopic hepatectomy were compared in different BMI stratification. We investigated overall survival and relapse-free survival across the BMI categories.

**Results:**

Of the 201 patients, 23 (11.44%) were underweight, 96 (47.76%) were normal weight, and 82 (40.80%) were overweight. The overall complication rate in the underweight group was much higher than that in the normal weight and overweight groups (*p*=0.048). Postoperative complications, underweight patients developed grade III or higher Clavien-Dindo classifications (*p*=0.042). Among the three BMI groups, there were no significant differences in overall and relapse-free survival with Kaplan-Meier analysis (*p*=0.104 and *p*=0.190, respectively). On the other hand, gender, age, liver cirrhosis, bile leak, ascites, and Clavien classification (III-IV) were not independent risk factors for overall and relapse-free survival in multivariable Cox proportional hazards models.

**Conclusions:**

BMI status does not affect patients with liver carcinoma long-term surgical outcomes concerned to overall survival and relapse-free survival after laparoscopic hepatectomy. However, being underweight was associated with an increased perioperative complication rate, and perioperative careful monitoring might be required after hepatectomy for underweight with liver carcinoma.

## Introduction

Liver carcinoma is one of the most malignant cancers worldwide and has a particularly high incidence rate in Asian countries. Liver resection is the main method used for the treatment of liver cancer, and the potential advantages of laparoscopy, such as minimally invasive technique, fewer complications, and shorter intraoperative hospital stay, have been identified [1, 2]. Although patients could get an early diagnosis combined with comprehensive treatment nowadays, due to the tumor’s malignant character, the prognosis of liver cancer remains unsatisfactory [3, 4]. Factors such as age, tumor size and tumor number, pathologic TNM stage, and vascular invasion have been identified as factors influencing the prognosis of liver cancer patients [5–7]. Several researchers have found that overweight status increases the risk of hepatocellular carcinoma (HCC) by 17%, and obese status increases the risk of HCC by 90% compared with normal-weight individuals [8, 9]. However, the relationship between weight and laparoscopic hepatectomy prognosis for liver carcinoma remains unclear.

Body mass index (BMI; kg/m^2^) is a convenient and simple surrogate measure of body fat distribution in clinical settings. The BMI values used to detect overweight and obese status recommended by the World Health Organization (>25 kg/m^2^) are higher than those suggested by Asian population-based studies (22-25 kg/m^2^) [10, 11]. The Working Group on Obesity in China has identified that a BMI of 23 is the most sensitive and specific indicator for the overweight status of Chinese people [12]. Therefore, it is useful to evaluate the association between the prognosis of liver carcinoma and overweight defined by this BMI value.

The prognosis of liver cancer remains unsatisfactory. Survival rates at 6, 12, and 24 months after the initial diagnosis have been reported to be 44.1%, 21.7%, and 14.2%, respectively [13]. Many studies have focused on the Child-Pugh score and the a-fetoprotein (AFP) concentration to determine the prognosis of liver cancer [14, 15]. However, the data concerning the influence of underweight and overweight status on this disease have not yet to be defined. The purpose of this study was to assess the effects of weight using BMI on relevant perioperative complications and the overall and relapse-free survival rates in patients with liver carcinoma who underwent laparoscopic hepatectomy.

## Methods

### Patients and diagnosis

A retrospective cohort study that spanned a 13-year period from August 2003 to April 2016 was performed. A total of 201 patients who underwent laparoscopic hepatectomy for liver carcinoma at our institution were identified. Institutional Review Board approval for this study was obtained from the Sir Run Run Shaw Hospital, School of Medicine, Zhejiang University.

Patients were divided into three groups by BMI according to the WHO’s definition of obesity restricted to the Asia-Pacific region: underweight < 18.5 kg/m^2^, normal weight 18.5–23 kg/m^2^, and overweight>23 kg/m^2^ [16]. BMI was calculated in line with the standardized definition as weight in kilograms divided by height in meters squared, and BMI was recorded on the morning of the surgery.

The diagnosis of liver carcinoma was established using imaging enhanced computed tomography (CT) or magnetic resonance imaging (MRI)) and pathology reports.

### Inclusion and exclusion criteria

LH was chosen as the initial therapy for all patients diagnosed with a liver tumor in the present study. The tumor size larger than 10 cm, with tumors invading major vessels, Child-Plug score worse than B, with tumor thrombus in the main portal vein were restricted to the procedure of laparoscopy. Patients who were younger than 18 years old, ASA > IV, or pregnancy were excluded from the study.

### Data collection

#### Surgical technique

Laparoscopic hepatectomy was performed as previously described [17]. Regional occlusion of the left/right inflow and outflow of the liver instead of total hepatic vascular occlusion was used to minimize liver ischemia/reperfusion injury [16]. In the early years of the study (2003–2006), parenchymal transection of the liver was achieved with the LPMOD (Peng’s multifunction operative dissector, SY-IIIB, Hangzhou ShuYou Medical Equipment Co., Ltd, China) technique. Since 2007, most cases have been performed using an ultrasonic aspirator (CUSA; Valleylab, Boulder, Colo). If there were unclear tumor margins, uncontrolled bleeding, embolism, severe adhesion, or other complications, the laparoscopic procedure was changed to open hepatectomy.

#### Follow-up and analysis

After being discharged from the hospital, all patients were followed up monthly in the first year. The follow-up included physical examinations, a computed tomographic scan or magnetic resonance imaging scan, and alpha-feto-protein (AFP) measurements. If no recurrence was detected, we extended the follow-up time to a quarter. Recurrence was defined as new typical features of a mass on imaging or a rising AFP level. A biopsy was performed if necessary. Survival was defined as the interval from the date of diagnosis of liver cancer to the date of death or the last visit before February 2019.

All patients were followed up in February 2019, and the median follow-up duration was 27 months (range, 2 months).

### Data analysis

We used the Shapiro–Wilk test to confirm the normality of data distribution. We presented continuous data as the mean (SD), and we presented categorical data as numbers or percentages. A one-way ANOVA combined with the Bonferroni test for post hoc testing was used in analyzing the continuous data. The chi-square or Fisher’s exact test combined with the Mann–Whitney *U* test for post hoc testing was used in analyzing the categorical data. The durations of overall survival and relapse-free survival were calculated by Kaplan-Meier analysis, and the results for the subgroups of patients were compared with the log-rank test. A *p* value < 0.05 was regarded as statistically significant. All statistical analysis was conducted with SPSS version 19.0.

## Results

### Patient-related variables

Table 1 shows the basic demographics of the patients with the liver disease for the three groups. A total of 201 patients were included in the study. Among them, there were 23 (11.4%) underweight, 96 (47.8%) normal weight, and 82 (40.8%) overweight patients. The overweight group had significant diabetic complications (*p*<0.01). Patient characteristics, including age, sex, Child-Pugh score, American Society of Anesthesiologists (ASA) score, hypertension, pulmonary, cardiovascular, and cerebrovascular were not significantly different between the three groups, except for liver cirrhosis (*p*<0.005), were not significantly different between the three groups. But concerning to the cirrhosis, the overweight patients had a significant occurrence(*p*<0.05).

**Table 1:** Patient Demographics with liver cancer stratified by BMI status

Variables	Underweight (*N*=23)	Normal weight (*N*=96) *N*=201	Overweight (*N*=82)	*p* value
Age (years)^a^	55.96±18.78	57.08±12.11	58.17±12.49	0.736
Gender				
Male	14 (60.9)	75 (78.1)	62 (75.6)	0.226
Female	9 (39.1)	21 (21.9)	20 (24.4)	
ASA score				
1–2	20 (87.0)	83 (86.5)	69 (84.1)	0.891
3–4	3 (13.0)	13 (13.5)	13 (15.9)	
Diabetes mellitus	2 (8.7)	2 (2.1)	15 (18.3)	<0.01^*^
Hypertension	1 (4.3)	8 (8.3)	10 (12.2)	0.46
Pulmonary comorbidity	1 (4.3)	11 (11.5)	3 (3.7)	0.45
Cardiovascular	1 (4.3)	2 (2.1)	3 (3.7)	0.76
Cerebrovascular	1 (4.3)	1 (1.0)	0 (0)	0.18
Child-Pugh score				
Class A	22 (95.7)	86 (89.6)	71 (86.6)	0.457
Class B	1 (4.3)	10 (10.4)	11 (13.4)	
Liver cirrhosis	3 (15)	48 (100)	32 (54)	0.005^*^
Album g/dl	3.86±0.49	4.04±0.50	3.98±0.59	0.385

*ASA* American Society of Anesthesiologists, ^*^p<0.05

### Tumor-related variables

Table 2 displays the oncological status, including tumor size, *tumor* number, *tumor* stage, the extent of live resection, and UICC. The oncological status was comparable in the three BMI groups.

**Table 2:** Oncological status stratified by BMI status

Variables	Underweight *N*=23	Normal weight *N*=96	Overweight *N*=82	*p* value
Tumor size	4.04±2.43	3.91±2.03	3.53±1.94	0.377
Tumor number	1.43±1.04	1.23±0.59	1.21±0.60	0.330
Tumor				
Cholangiocarcinoma	0 (0)	6 (6.3)	3 (3.7)	0.389
Hepatocellular carcinoma	18 (78.3)	77 (80.2)	68 (83.0)	
Adenocarcinoma	3 (13.0)	5 (5.2)	2 (2.4)	
Metastatic hepatic	2 (8.7)	8 (8.3)	9 (11.0)	
UICC				
Stage I	8 (34.8%)	34 (35.4%)	27 (32.9%)	0.822
Stage II	7 (30.4%)	46 (47.9%)	38 (46.3%)	
Stage IIIA	2 (8.7%)	10 (10.4%)	13 (15.9%)	
Stage IIIB	4 (17.4%)	5 (5.2%)	4 (4.9%)	
Stage IIIC	2 (8.7%)	1 (1.0%)	0 (0)	
Extent of live resection				
Wedge	4 (17.4)	17 (17.7)	22 (26.8)	0.555
Segmentectomy	15 (65.2)	59 (61.5)	48 (58.5)	
Hemihepatectomy	4 (17.4)	20 (20.8)	12 (14.6)	

*UICC* Union for International Cancer Control, ^*^p<0.05

### Short-term outcomes

Table 3 shows short-term outcomes stratified by BMI status. The conversion rates were 13.0%, 14.6%, and 14.6% for the three BMI groups. The mean postoperative length of hospital stay for the three groups was 9.87±4.36 days, 10.10±5.88 days, and 9.82±5.27 days, *p*= 0.938, respectively. Interestingly, the overall complication rate in the underweight group was much higher than that in the normal weight and overweight groups (47.82% vs 21.88% vs 17.10%, respectively, *p*=0.048). Regarding postoperative complications, underweight patients developed grade III or higher Clavien-Dindo classifications more easily than patients in the other two groups (*p*=0.042). The underweight group had significantly higher comorbidity rates (*p*=0.048) of bile leak and ascites (*p*=0.037 and *p*=0.032, respectively) than the normal weight group. Other variables, including estimated blood loss and blood transfusion, as well as postoperative complications, intraabdominal sepsis, surgical site infection, pneumonia, bleeding, and chemotherapy.

**Table 3:** Short-term outcomes stratified by BMI status

Variables	Underweight	Normal weight	Overweight	*p* value
Estimated blood loss (ml, mean ± SD)	635.65±605.51	468.48±760.08	426.46±634.45	0.444
Blood transfusion	178.26±285.97	198.13±493.80	152.56±368.28	0.777
Conversion	3 (13.0)	14 (14.6)	12 (14.6)	0.980
Operative time (min, mean ± SD)	177.04±110.10	171.24±104.10	159.93±79.50	0.641
Postoperative length of stay (D)	9.87±4.36	10.10±5.88	9.82±5.27	0.938
Complication	11 (47.8)	21 (21.9)	14 (17.1)	0.048^*^
Bile leak	5 (21.7)	6 (6.3)	4 (4.9)	0.037^*^
Introabodominal sepsis	1 (4.3)	4 (4.2)	3 (3.7)	0.432
Surgical site infection	0 (0)	3 (3.1)	1 (1.2)	0.737
Ascites	3 (13.0)	4 (4.2)	0 (0)	0.032^*^
Pneumonia	2 (8.7)	3 (3.1)	3 (3.7)	0.758
Bleeding	0 (0)	1 (1.0)	3 (3.7)	0.647
Clavien classification				
I-II	5 (21.7)	18 (18.8)	15 (18.3)	0.732
III–IV	2 (8.7)	3 (3.1)	1 (1.2)	0.042*
Chemotherapy	7 (30.4)	38 (39.6)	38/82 (46.3)	0.442

^*^p<0.05

### Long-term outcomes

**Fig. 1:**
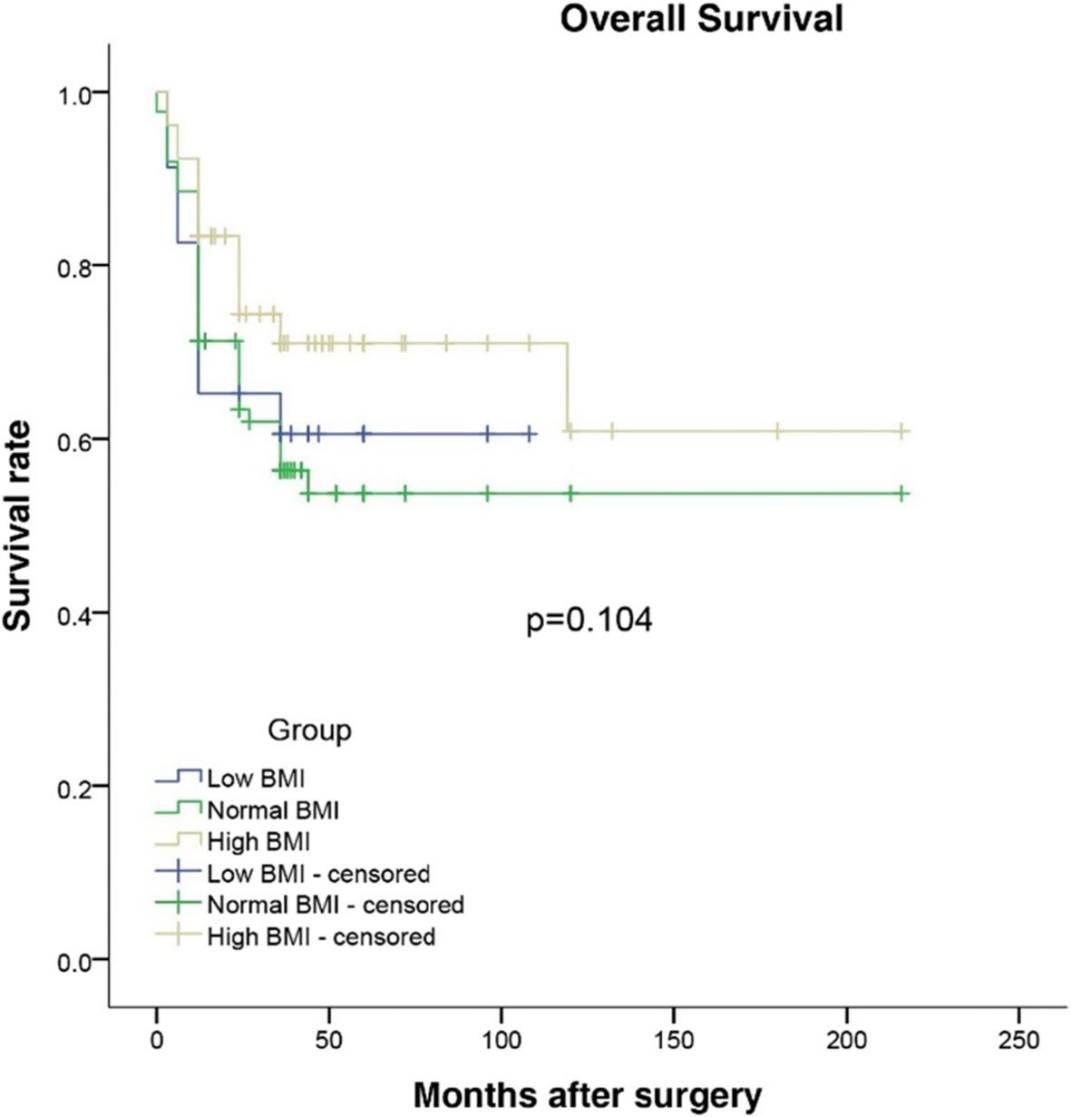
Overall survival after laparoscopic hepatectomy for patients with liver cancer stratified by BMI status

**Fig. 2:**
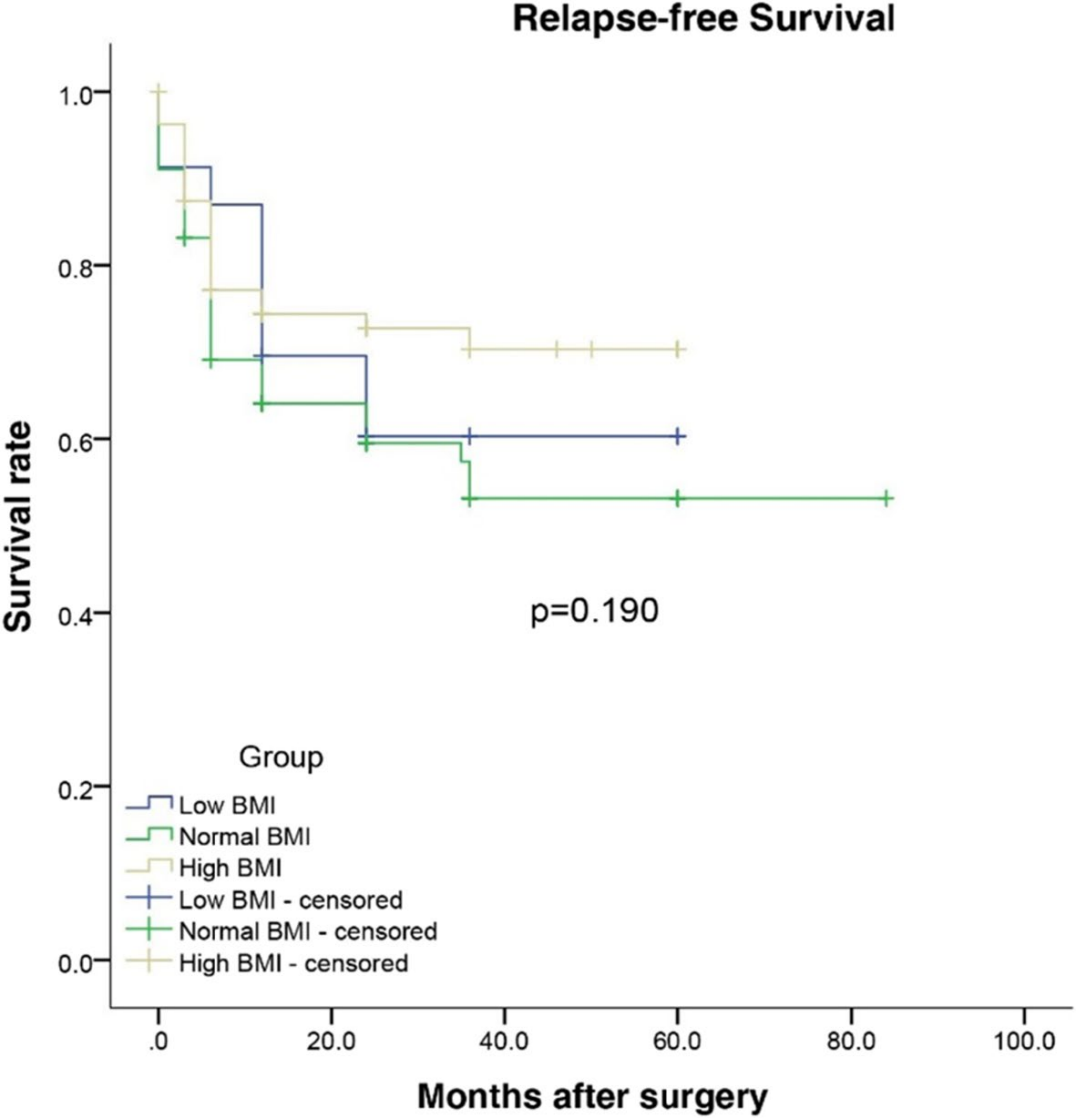
Relapse-free survival after laparoscopic hepatectomy for patients with liver cancer stratified by BMI status

The median follow-up durations were 26 months, 30 months, and 28 months in the underweight, normal weight, and overweight groups, respectively. Kaplan-Meier survival curve analysis of overall survival rate and relapse-free survival rate in patients stratified by the BMI are shown in Figs. 1 and 2. Overall and relapse-free survival showed no significance among the three groups (*p*=0.104 and *p*=0.190, respectively). The variables that have important clinical significance were incorporated into the multivariate analysis. The results revealed that gender, age, liver cirrhosis, bile leak, ascites, and Clavien classification (III-IV) were not independent risk factors for overall and relapse-free survival. So maybe preoperative nutritional supplementation could improve outcomes, as was done in the ERAS setting.

## Discussion

Laparoscopic hepatectomy has become a feasible option for patients with liver malignancy. As it is a new technique, researchers have given more attention to perioperative and long-term outcomes. Although being overweight and obese does not preclude laparoscopy [16], little is known about the influence of recipient BMI on long-term outcomes, especially the recurrence rate and the overall survival rate after LH for HCC. In the present study, we observed that patients with elevated BMI accounted for 40.8%. General surgeons will encounter more overweight and obese patients with liver cancer in the future [18]. Therefore, it is important to fully understand the effect of elevated BMI on these patients. We carried out this retrospective analysis using our institutional database and showed that (i) being overweight did not increase the conversion rate, (ii) being underweight was associated with an increased number of complications and more severe complications, and (iii) being overweight does not affect patients with liver carcinoma surgical outcomes after laparoscopic hepatectomy. It was interesting to find that being overweight is not an independent risk factor for overall and relapse-free survival for liver carcinoma patients.

Many studies have demonstrated that increased BMI increases the laparoscopic conversion rate and prolongs the operative time [19, 20]; thus, surgeons tend to be reluctant to perform laparoscopic surgery for overweight patients. However, there were no differences in the conversion rate and operative time across the three groups in our study, which was consistent with Troisi et al. [21]. This may be because all operations were performed by the same experienced surgeon team who had finished training and was capable of removing liver tumors using laparoscopic techniques, and even more, there were no patients with BMI greater than 40 in the present study. Meanwhile, not in line with previous studies, which found that overweight patients had worse outcomes than their leaner counterparts [22], our results showed that overweight patients had a lower complication rate (17.10%) compared to underweight (47.82%) and normal-weight patients (21.88%) and that decreased BMI values were a risk factor for a higher incidence of postoperative complications. We also found that severe complications of grades III to IV according to the Clavien-Dindo classification were much more frequent in the underweight group than in the normal weight and overweight group. One cause may be that overweight patients are protected by adequate fat storage, better nutrition, and systemic insulin resistance which underweight people do not have [23]. Meanwhile, patients with a lower BMI preoperatively are more likely to indicate excessive nutritional consumption and malnutrition resulting from the more aggressive tumor [24–26].

To our knowledge, little research has investigated the association between BMI and prognosis in patients undergoing LH with liver cancer. Daniel reported that overweight status had better oncologic outcomes following hepatectomy in HCC patients [27], but the endpoint of Daniel’s study was 3 months. The follow-up duration was relatively short, and more data were needed on the overall survival rate as an important therapeutic measure of liver cancer if treatment intensity and life expectancy were to be judged [15]. Chronic disorders such as cardiovascular disease, hypertension, and diabetes, which are closely related to obesity, increase the risk of physical disability and mortality rates in the long run [28]. It has been reported that nearly 70% of deaths related to high BMIs are due to cardiovascular disease, and over 60% of those deaths occur among obese patients [29]. Our study aimed to verify the long-term outcome of liver carcinoma patients who underwent LH, and our results suggested that BMI status had no impact on either overall or relapse-free survival in univariate and multivariate analysis. Therefore, the influence of BMI on survival for liver carcinoma patients who underwent LH remains controversial; thus, multicenter and large sample studies were future needed.

Moreover, as reported, postoperative complications had a negative impact on tumor recurrence and the long-term survival rate, especially for severe postoperative complications [30]. In our study, we again verified that postoperative complications might have an effect on the prognosis of liver cancer patients. We found that underweight patients suffered more complications. One reason is that the surgical trauma and tissue damage of the complications could result in immune suppression and, in turn increase the possibility of immune escape and tumor progression [31, 32]. Second, a large number of cytotoxic mediators from the inflammatory response caused by the infected complications could provide a microenvironment for the growth and invasion of tumor cells and further promote the development of tumor recurrence [33]. At the same time, minimally invasive laparoscopic surgery could avoid complications such as poor wound healing and pulmonary infection caused by a long incision, and it yielded more advantages for overweight patients.

This study has some limitations. First, it was a retrospective, non-randomized survey that could not been avoided selection bias. Due to few patients in the underweight group, type II error is likely to be the cause of stage III tumor in low-weight patients. Second, this was a single-center study, and there is still no standard treatment for liver cancer. The results in our center might have been influenced by our surgical strategy. Third, we only used BMI to assess overweight, which was inadequate for assessing the abdominal adiposity of Asian people.

Despite these limitations, our study helps to clarify that LH for the treatment of overweight patients with hepatocellular carcinoma is feasible and safe. These results need to be verified by larger prospective and randomized studies.

## Conclusion

Our data showed that BMI status does not affect patients with liver carcinoma long-term surgical outcomes concerned to overall survive and relapse-free survival after laparoscopic hepatectomy. Patients can be scheduled for surgery normally, regardless overweight and underweight. However, perioperative careful monitoring might be required for

## Data Availability

The datasets used and/or analyzed during the current study are available from the corresponding author on reasonable request

## Abbreviations

BMI: Body mass index
LH: Laparoscopic hepatectomy
ASA: American Society of Anesthesiologists
LPMOD: Peng’s multifunction operative dissector
CT: Enhanced computed tomography
MRI: Magnetic resonance imaging
AFP: Alpha-feto-protein
HCC: Hepatocellular carcinoma
